# Multimodal Imaging of Photoreceptor Structure in Choroideremia

**DOI:** 10.1371/journal.pone.0167526

**Published:** 2016-12-09

**Authors:** Lynn W. Sun, Ryan D. Johnson, Vesper Williams, Phyllis Summerfelt, Alfredo Dubra, David V. Weinberg, Kimberly E. Stepien, Gerald A. Fishman, Joseph Carroll

**Affiliations:** 1 Department of Ophthalmology & Visual Sciences, Medical College of Wisconsin, Milwaukee, Wisconsin, United States of America; 2 Department of Biomedical Engineering, Marquette University, Milwaukee, Wisconsin, United States of America; 3 Department of Cell Biology, Neurobiology & Anatomy, Medical College of Wisconsin, Milwaukee, Wisconsin, United States of America; 4 Department of Biophysics, Medical College of Wisconsin, Milwaukee, Wisconsin, United States of America; 5 The Chicago Lighthouse, Chicago, Illinois, United States of America; University of Miami, UNITED STATES

## Abstract

**Purpose:**

Choroideremia is a progressive X-linked recessive dystrophy, characterized by degeneration of the retinal pigment epithelium (RPE), choroid, choriocapillaris, and photoreceptors. We examined photoreceptor structure in a series of subjects with choroideremia with particular attention to areas bordering atrophic lesions.

**Methods:**

Twelve males with clinically-diagnosed choroideremia and confirmed hemizygous mutations in the *CHM* gene were examined. High-resolution images of the retina were obtained using spectral domain optical coherence tomography (SD-OCT) and both confocal and non-confocal split-detector adaptive optics scanning light ophthalmoscope (AOSLO) techniques.

**Results:**

Eleven *CHM* gene mutations (3 novel) were identified; three subjects had the same mutation and one subject had two mutations. SD-OCT findings included interdigitation zone (IZ) attenuation or loss in 10/12 subjects, often in areas with intact ellipsoid zones; RPE thinning in all subjects; interlaminar bridges in the imaged areas of 10/12 subjects; and outer retinal tubulations (ORTs) in 10/12 subjects. Only split-detector AOSLO could reliably resolve cones near lesion borders, and such cones were abnormally heterogeneous in morphology, diameter and density. On split-detector imaging, the cone mosaic terminated sharply at lesion borders in 5/5 cases examined. Split-detector imaging detected remnant cone inner segments within ORTs, which were generally contiguous with a central patch of preserved retina.

**Conclusions:**

Early IZ dropout and RPE thinning on SD-OCT are consistent with previously published results. Evidence of remnant cone inner segments within ORTs and the continuity of the ORTs with preserved retina suggests that these may represent an intermediate state of retinal degeneration prior to complete atrophy. Taken together, these results supports a model of choroideremia in which the RPE degenerates before photoreceptors.

## Introduction

Choroideremia is a progressive X-linked recessive dystrophy characterized by degeneration of the sensory retina[1] secondary to *CHM* gene mutations.[2, 3] Affected males typically experience nyctalopia and peripheral visual field loss by the second decade of life; central vision is usually affected later in the course of the disease.[4] Early peripheral pigmentary mottling of the fundus develops into choroidal and retinal atrophy, typically beginning in the midperiphery and expanding both centrally and peripherally.[5] Previous histological and optical coherence tomography (OCT) studies have led to various models of degeneration, including 1) a diffuse and independent degeneration of the choriocapillaris, retinal pigment epithelium (RPE), and neural retina,[6, 7] 2) a primary photoreceptor degeneration followed by RPE and choroidal atrophy,[6, 8] and 3) a primary RPE degeneration followed by photoreceptor loss and choroidal atrophy.[9–11] Additional OCT-documented characteristics of choroideremia include the presence of interlaminar bridges[8] (ILBs, wedge-shaped hyporeflective structures bridging the inner and the outer retina) and outer retinal tubulations[4, 12] (ORTs, structures in the outer retina composed of deteriorating photoreceptors and remnant external limiting membrane (ELM) that typically appear on OCT as hyperreflective ovaloid structures with a hyporeflective lumen[13]). Finally, Lazow *et al* report a relatively abrupt termination of the inner segment/outer segment ellipsoid zone (EZ) and ELM that frequently coincide with ILBs.[9, 14] While these studies highlight the utility of commercially-available SD-OCT in assessing the layered macroanatomy of the retina, their limited resolution precludes direct examination of photoreceptor structure on a cellular scale.

By correcting for the eye’s monochromatic aberrations, confocal adaptive optics scanning light ophthalmoscopy (AOSLO) enables cellular-resolution imaging of photoreceptor structure,[15, 16] and numerous studies have used AOSLO to probe the fine anatomy of retinal degenerations.[12, 17–26] Indeed, confocal AOSLO has recently been used to document lack of cone reflectivity in the ORTS found in age-related macular degeneration.[27] In choroideremia, AOSLO imaging has uncovered novel pathologic features including bubble-like lesions that appear to co-localize to structures in the choroid visualized by SD-OCT[26] and yielded results suggesting a primary RPE degeneration with subsequent loss of photoreceptors and choroid.[12, 26] Recent work by Morgan *et al* showed an abrupt decline in confocal AOSLO photoreceptor reflectivity in areas of RPE degeneration[26], while SD-OCT imaging revealed interdigitation zone (IZ, thought to represent the junction of photoreceptors and RPE) dropout and RPE thinning prior to disruptions in the overlying EZ.[26] Together, these results may imply cone loss in choroideremia is secondary to the atrophy of supporting RPE. However, imaging of cone structure with confocal AOSLO imaging relies upon propagation and reflectance of the incident imaging light within an intact and properly aligned outer segment (i.e., waveguiding).[28] Disruptions of cone morphology in choroideremia[6, 7] might therefore interfere with confocal imaging of cones, making it impossible to distinguish a non-waveguiding cone from an absent one. Thus, within the context of choroideremia, it remains unknown whether the photoreceptor mosaic truly terminates at the point of RPE atrophy.

In contrast, the signal in split-detector AOSLO originates from multiply-scattered light from the inner segment,[28] thus decoupling cone visualization from outer segment morphology. Split-detector AOSLO has been used to provide unambiguous characterization of remnant cone morphology in a number of retinal diseases.[28–33] Here, we used split-detector AOSLO in conjunction with confocal AOSLO and SD-OCT to assess outer retinal structure in choroideremia. We examined areas bordering regions of atrophy and within characteristic ORTs, ILBs, and bubble-like lesions with the aim of clarifying the sequence of degeneration, which may in turn assist the development of therapeutic strategies.[34, 35]

## Methods

### Subjects

Research procedures were in accordance with the tenets of the Declaration of Helsinki and were approved by the Institutional Review Boards of the Medical College of Wisconsin and the Children’s Hospital of Wisconsin. After explanation of the nature and possible consequences of the study, informed written consent was obtained from all subjects. Subjects with clinically-diagnosed choroideremia were eligible for inclusion. Between August 2010 and June 2014, 12 male subjects were enrolled in the study and imaged (**Table 1**); several subjects were followed across more than one imaging session, and the most recent follow-up imaging was performed in January 2016. For comparison, we utilized images from a healthy subject (28-year-old male, JC_0616) as well as previously-published cone density data from nine healthy subjects.[36]

**Table 1:** Characteristics of 12 Male Subjects with Choroideremia.

Subject	Age	Identified *CHM* Gene Mutations	Genetic References	Eye	Near-Foveal Cones/mm^2^ ^‡^	Central RT	Average RT	BLLs	ILBs	ORTs
KS_0044	19	Deletion of exons 6, 7, and 8	Freund PR et al, Mol Genet Genomic Med 2016	OD	87,603	99% (+)	Normal	Yes	Yes	Yes
JC_0618*	46	c.757C>T; p.Arg253Stop	Van den Hurk JA et al, Hum Genet 2003	OD	N/A^§^	Normal	Normal	Yes	Yes	Yes
JC_0621	64	Deletion of exon 15	Novel exon deletion	OS	67,886	Normal	Normal	No	Yes	Yes
JC_0672	55	Partial deletion of exon 4 c.314+10127T>A splice site mutation	McTaggart et al, Hum Mutat 2002 Van den Hurk JA et al, Hum Genet 2003	OD	N/A^§^	Normal	99% (-)	Yes	Yes	Yes
JC_0675	56	c.190-2A>G splice site mutation	Van den Hurk JA et al, Hum Genet 2003	OS	N/A^§^	99% (-)	99% (-)	Yes	Yes	Yes
JC_0699	72	c.1349G>A; p.Arg450Lys	Reported to the Vision Variation Database[37]	OD	N/A^§^	Normal	Normal	Yes	No	Yes
JC_0752	29	c.1530_1531insA fs*4; p.Thr511Asn fs*4	Novel frameshift with truncation	OD	N/A^§^	Normal	Normal	Yes	Yes	Yes
JC_0754	40	c.1579_1582delTTGT; p.Leu527His fs*7	Novel frameshift with truncation	OD	71,890	99% (+)	Normal	Yes	Yes	Yes
JC_0778^†^	22	c.757C>T; p.Arg253Stop	Van den Hurk JA et al, Hum Genet 2003	OD	95,771	95% (+)	Normal	Yes	Yes	Yes
JC_0782^†^	25	c.757C>T; p.Arg253Stop	Van den Hurk JA et al, Hum Genet 2003	OD	76,551	Normal	Normal	No	No	No
JC_0942	40	c.1144G>T; p.Glu282Stop	Reported to the Leiden Open Variation Database[38]	OD	N/A^§^	95% (+)	Normal	Yes	Yes	Yes
DW_10173	40	Deletion of exons 1–15 (complete deletion)	Den Dunnen and Antonarakis, Hum Genet 2001	OS	N/A^§^	Normal	95% (-)	Yes	Yes	Yes

Eye = eye used for all measurements, assessments, imaging, and analysis. Central RT = central retinal thickness as a percentile of normal. Average RT = average retinal thickness as a percentile of normal. BLLs = bubble-like lesions. ILBs = interlaminar bridges. ORTs = outer retinal tubulations. Fs = frameshift. Del = deletion. Ins = insertion. Stop = stop codon (truncation). *(*n*) = stop codon in *n* residues.

* Unrelated to JC_0778 and JC_0782.

† First cousins. JC_0782 independently sought genetic testing and presented results at time of study.

‡ Cone densities were measured at 0.70° (202.5 μm) eccentricity. Normal mean cone density at this eccentricity is 76,038 cones/mm^2^ (SD 31,543 cones/mm^2^).

§ In subjects with no EZ band visible at the fovea on SD-OCT due to advanced pathology, no near-foveal cone count was measured.

#### Genetic Testing and Predictions

Whole blood was collected for analysis from 11 out of 12 subjects; the 12^th^ subject had previously obtained genetic testing. Eight samples were sent to The John and Marcia Carver Nonprofit Genetic Testing Laboratory (University of Iowa, IA, USA), two to the National Ophthalmic Disease Genotyping and Phenotyping Network (EyeGENE, National Eye Institute, Bethesda, MD, USA), and one to the Casey Eye Institute Molecular Diagnostics Laboratory (Oregon Health & Science University, OR, USA). Each sample was screened for mutations in the *CHM* gene. For the remaining subject, genetic records were available from the referring physician (The Chicago Lighthouse, Chicago, IL, USA). Amino acid changes due to novel gene mutations were determined using Mutation Taster (available in the public domain at http://www.mutationtaster.org).

#### Clinical Examination

Dilated fundus exams were performed prior to imaging. Axial length measurements were obtained using an IOL Master (Carl Zeiss Meditec, Dublin, CA USA) and used to calibrate the lateral scale of all retinal images (see Methods). For all imaging sessions, one eye of each subject (OD in all except JC_0675 and DW_10173) was dilated and accommodation suspended using one drop each of phenylephrine (2.5%) and tropicamide (1%). Color fundus images were obtained in all subjects using a Zeiss VisuCam 200NM (Carl Zeiss Meditec, Dublin, CA, USA) and/or an OPTOS Ultra-Widefield fundus camera (Optos plc, Dunfermline, Scotland, United Kingdom). Fundus images were exported using onboard software for subsequent alignment with high-resolution images.

#### Spectral Domain Optical Coherence Tomography (SD-OCT)

Volumetric images of the macula were obtained using optical coherence tomography (Cirrus HD-OCT; Carl Zeiss Meditec, Dublin, CA, USA). Volumes were nominally 6 x 6 mm and consisted of 128 B-scans (512 A-scans/B-scan). Retinal thickness was calculated using the built-in macular analysis software (version 5.0) as the distance between inner limiting membrane (ILM) and RPE boundaries. Additional high-density volume scans of the macula were acquired (Envisu 2300; Bioptigen, Research Triangle Park, NC, USA); scans were nominally 6 x 6 mm or 7 x 7 mm in size and consisted of 250 B-scans (700 A-scans/B-scan). High-density line scans (nominal scan length = 6 or 7 mm; 1000 A-scans/B-scan, 100 to 120 repeated B-scans) were acquired both horizontally and vertically through the foveal center. Line scans were registered and averaged to reduce image speckle as previously described.[39] In a few cases, 3 to 4 closely spaced B-scans within a volume were averaged to generate higher signal to noise images at non-foveal regions of interest (ROIs). Assessment of layer integrity at specific locations was performed using the longitudinal reflectivity profile (LRP), as previously described.[40, 41] Four layers were manually identified in the resultant LRPs: the external limiting membrane, ELM; the EZ; the IZ; and the retinal pigment epithelium, RPE.

Using custom designed Java (Oracle, Redwood City, CA) software, we derived *en face* summed volume projection (SVP) images from the Bioptigen SD-OCT volumes of 8 subjects using a previously described method.[31, 42] These images were created to visualize the two-dimensional layout of ORTs.

#### Adaptive Optics Scanning Light Ophthalmoscopy

Reflectance confocal images of the photoreceptor mosaic were obtained using a previously described AOSLO.[15, 43, 44] Retinal images were obtained using 775, 790, or 796 nm superluminescent diodes, subtending 1, 1.5, or 1.75° square fields of view. In 5 subjects (KS_0044, JC_0699, JC_0778, JC_0782, DW_10173), split-detector AOSLO images were captured simultaneously with confocal ones (and thus are in perfect spatial co-register). Foveal images were acquired by instructing the patient to fixate on the corners or edges of the imaging raster (eccentricities of 0.5 or 0.65°), while parafoveal images were acquired using an adjustable external fixation target extending out to eccentricities of up to 12°. Additional images were acquired as needed to assess specific ROIs.

Raw AOSLO retinal images were corrected for distortions, registered, and averaged as previously described.[32, 45] These registered and averaged AOSLO images were manually montaged using Adobe Photoshop (Adobe Systems, Inc., CA) as previously described.[32] Line scanning ophthalmoscope (LSO) images from the Cirrus HD-OCT and volume intensity projection images from the Bioptigen SD-OCT were exported and, together with AOSLO montages, aligned to the clinical fundus images. The relative sizes of multimodal images were derived by scaling the visual angle for an ideal eye with an axial length of 24 mm, 291 μm/degree, in proportion to the subject’s measured axial length.[46] Alignment was performed manually utilizing blood vessel patterns. This multimodal aligned overlay enabled comparison of retinal structure at specific ROIs.

For clarity, areas of retina (typically central) with discernible IZ and/or EZ structure will henceforth be referred to as “preserved retina,” while areas of retina (typically peripheral) with neither IZ nor EZ structure is referred to as “atrophic retina.” Regions of abnormal preserved retina that border atrophic lesions (generally within a few hundred microns) are referred to as “border regions.”

#### Parafoveal Cone Density Measurements and Statistical Analysis

**Fig 1:**
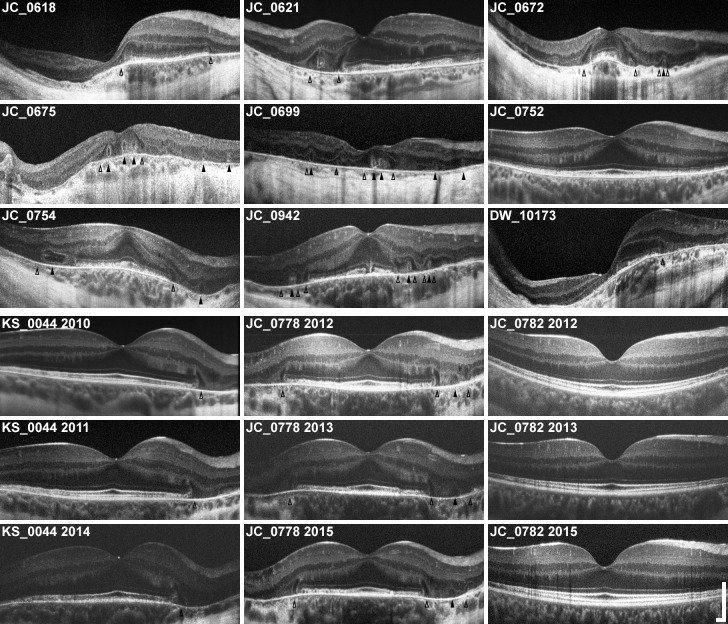
Horizontal SD-OCTs of all 12 subjects with X-linked choroideremia. Horizontal SD-OCT line scans through the fovea of all 12 subjects (labeled) are presented here in the same order given in **Table 1**. Images are scaled and cropped to subtend a uniform retinal distance (6 mm). Degree of pathology varies, and older patients generally exhibit greater loss of central retina. Pathologic features can be seen: outer retinal tubulations (ORTs) are visible as hyperreflective ovaloid rings with hyporeflective lumens (**filled arrowheads**) and interlaminar bridges (ILBs) are visible as wedge-shaped hyporeflective structures, sometimes with a hyperreflective exterior, extending from the outer plexiform layer to Bruch’s membrane (**open arrowheads**). ILBs frequently coincide with the termination of the central zone of preserved retina, and sometimes flank ORTs. Note the asymmetry of many OCTs, particularly in cases of severe pathology. Longitudinal images are shown for 3 subjects (KS_0044, JC_0778, and JC_0782), showing early IZ attenuation and characteristic peripheral-to-foveal progression of atrophy. **Scale bars, axial & lateral:** 250 μm.

In 5 subjects (KS_0044, JC_0621, JC_0754, JC_0778, and JC_0782) in whom OCT B-scans showed an intact EZ at the fovea, near-foveal cone densities were counted from AOSLO confocal images acquired on the same day as the OCT scans shown in **Fig 1**. Please note that despite overlap (KS_0044, JC_0778, and JC_0782), these are not the same five subjects for which AOSLO split detector imaging was obtained. Although JC_0752 had an intact EZ at the fovea, poor foveal image quality precluded cone counting. The anatomic fovea can sometimes be difficult to resolve due to extremely tight cone packing. Ability to visualize cones may also be locally or globally compromised on AOSLO despite high-quality OCT imaging, particularly in subjects with poor fixation, severe visual impairment, advanced age, and/or other media-opacifying intraocular processes (e.g. cataracts). ROIs used in cone counts were generated by manually cropping 50 x 50 μm square sampling areas at an average of 0.70° (approximately 202.5 μm) eccentricity. Cones were frequently not countable at the true anatomic foveal pit because such cones were too tightly packed to be resolvable on AOSLO.

Cones were identified from confocal AOSLO images using a previously-described custom semi-automated algorithm that locates cones based on local intensity maxima, followed by manual adjustments by a single observer (L.W.S.).[47] Image contrast was adjusted as needed to assist in manually identifying dimly reflective cones. Density (in cones/mm^2^) was calculated by dividing the number of counted cones by the summed area (in mm^2^) of the bound Voronoi domains in the ROI,[48] and compared to an exponential fit to normative cone density data from 9 healthy subjects developed by Wilk *et al*.[36] *T*-scores were calculated using Student’s *t*-test for two independent means, and corresponding two-tailed *p*-values were generated using *t*-scores and degrees of freedom calculated as independent samples (QuickCalcs, GraphPad Software, Inc., La Jolla, CA).

#### Cone Density and Diameter Measurements in Areas Bordering Lesions

Lesion borders were mapped using OCT *en face* imaging and projected onto confocal AOSLO images using the aforementioned registration methods. In subjects with split-detector AOSLO imaging, lesion borders were mapped using split-detection. Cone density near the borders of lesions was also measured using the split-detector AOSLO images because confocal AOSLO was unable to unambiguously identify all cones due to irregular reflectivity in pathologic areas. Paired 50 x 50 μm ROIs were cropped from each AOSLO montage of the 5 subjects with both split-detector and confocal imaging. ROI pairs were chosen such that each was within 200 μm of the nearest lesion border, and both were at approximately the same eccentricity from the foveal pit. One ROI was cropped from an area of visibly higher cone density and one was cropped from an area of visibly lower cone density. No semiautomated algorithm was available at the time of these experiments, so all cones were manually identified within each ROI by a single observer (L.W.S.). Because ROIs varied in eccentricity based on individual pathology, it was not possible to conduct formal statistical analyses on the data. However, comparisons to normal cone densities from the previously-referenced database of nine healthy subjects[36] were performed by using an exponential fit to normative cone density data to calculate expected differences in cone density at each of the pairs of ROIs sampled.

## Results

### Subject Demographics, Clinical Data, and Genetics

Subject demographics, genetic profiles, and a brief summary of OCT and AOSLO findings are given in **Table 1**. Twelve males (mean ± SD age = 44.6 ±16.5 years) with choroideremia were recruited based on clinical diagnoses of choroideremia, which took into account personal ocular history, presenting symptoms, and family history. Disease was confirmed by genetic analysis in 11 of 12 cases; in the 12^th^ case, JC_0782, the subject had obtained genetic testing through his referring physician prior to our study. This patient is also the first cousin of JC_0778, who was among the 11 patients for whom we requested genetic testing.

We identified a total of 11 mutations within the 12 subjects (**Table 1**), 8 of which had been previously reported. Two previously documented[49, 50] mutations, a splice site mutation and a partial deletion, were found in subject JC_0672. Three subjects were found to have the same previously published point mutation resulting in truncation, c.757C>T (p.Arg253Stop);[50] two of these subjects were first cousins (JC_0778 and JC_0782), but the third (JC_0618) was unrelated. Five other previously-reported mutations were found in our subjects: one complete *CHM* gene deletion, two deletions of one or more exons of the *CHM* gene, one single base pair substitution resulting in truncation, and one splice site mutation. To the best of our knowledge, the remaining 3 mutations were novel: a deletion of most or all of exon 15 (JC_0621) and two frameshift mutations (JC_0752 and JC_0754, both with truncation occurring shortly after frameshift). Because all novel mutations found in our subjects were deletions or frameshifts, the commonly-used tools SIFT, PROVEAN (both available in the public domain at http://provean.jcvi.org/index.php), and/or PolyPhen-2 2.2.2 (available in the public domain at http://genetics.bwh.harvard.edu/pph2) could not be used to assess probable pathogenicity.

### Pathologic Features Visualized with SD-OCT

**Fig 1** shows averaged horizontal line scans through the anatomic foveal pit of all subjects, and **S1 Fig** shows corresponding averaged vertical line scans also through the fovea. Upon examination of all horizontal and vertical line scans, the IZ band is absent and/or indistinct from the RPE band in all but two subjects even in areas where the outer retinal layers were otherwise visible, which is consistent with previous reports in choroideremia.[26] The exceptions were JC_0782 and KS_0044, both young subjects followed for extensive periods (JC_0782: age 21 at initial imaging, followed over 47 months; KS_0044: age 16 at initial imaging, followed over 36 months). In both cases, an intact and distinct IZ band was initially seen centrally with peripheral loss of IZ band discrimination. While JC_0782 has exhibited slow disease progression limited to the periphery, KS_0044 has progressed more rapidly with patchy IZ band loss first noted centrally at age 17 (6 months after initial visit) and near-complete loss of IZ band discrimination in both eyes at his latest visit (age 19). Repeat SD-OCTs taken at 3 different time points in 3 subjects (KS_0044, JC_0778, and JC_0782) are representative of the peripheral-to-central progression of the disease observed in the 7 subjects (KS_0044, JC_0618, JC_0621, JC_0699, JC_0752, JC_0778, and JC_0782) who were imaged across at least two visits.

In all subjects, areas of thinned RPE band were also noted in the periphery, and sometimes in the fovea. In general, RPE thinning is associated with notable overlying abnormalities of the IZ and EZ bands. Finally, loss of the EZ band was also noted peripherally in all subjects except JC_0782, who exhibited patchy EZ band attenuation at the far periphery of the 7 mm area examined by SD-OCT line scan. In 10/12 cases, both the EZ band and overlying ELM were noted to terminate sharply at an ILB in at least one hemiretina, again consistent with previous reports.[9]

Other previously reported SD-OCT features were also seen in our subjects, including relative retention of retinal lamination at and around the fovea, chorioretinal atrophy in the periphery, ORTs, and ILBs. Changes in retinal thickness were also observed, including central thickening in 4 patients (aged 19, 22, 40 and 40 years) and general thinning in 3 patients (aged 40, 55, and 56 years). **Table 1** summarizes these pathologic SD-OCT findings. Note, not all findings are evident in the horizontal and vertical line scans through the fovea; some are visible only in volume scans through the 7 mm foveal area.

### Parafoveal Cone Density Measurements using Confocal AOSLO

Within our cohort of 12 subjects, cone densities were measured at or near the anatomic foveal pit in 5 subjects. Cone densities were only obtained from subjects with OCT B-scans showing intact EZs at the fovea. This is because only confocal imaging could resolve foveal cones, and only reflectivity in areas with intact EZs could be confidently regarded as cone reflectivity could be used for cone density measurements. Consequently, cone densities were only measured in subjects with comparatively mild pathology. Confocal AOSLO images acquired on the same day as the OCT B-scans shown in **Fig 1** were used in near-foveal cone density measurements. Sampling windows for these 5 subjects are shown in **S2 Fig**. The average eccentricity of the 5 near-foveal cone density measurements was 202.5 μm, or approximately 0.70°. The mean (±SD) near-foveal cone density was 79,940 ± 11,521 cones/mm^2^ (**Table 1**), which did not differ significantly (two-tailed *p* = 0.79) from the normal mean (±SD) near-foveal cone density at 202.5 μm eccentricity, 76,038 ± 31,542.75 cones/mm^2^.[36]

### Disambiguation of Remnant Cone Structure with Split-Detector AOSLO

**Fig 2:**
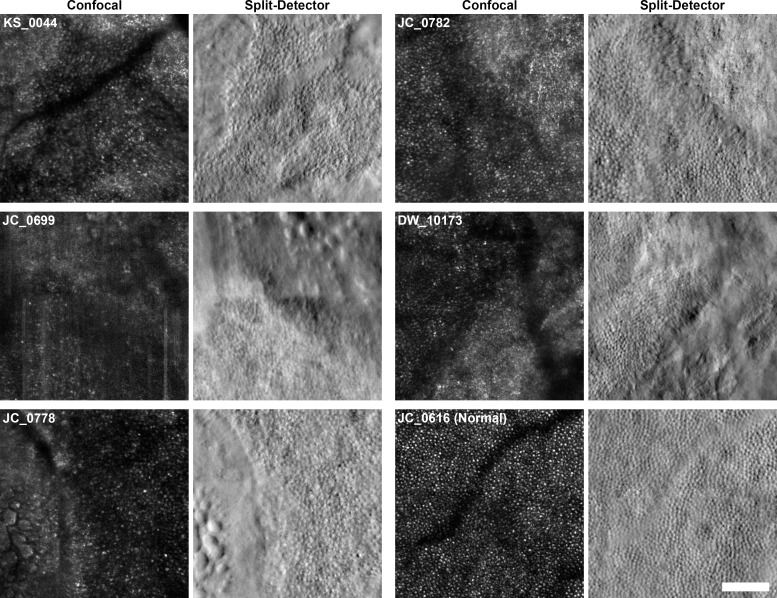
Split-detector AOSLO imaging allows unambiguous imaging of photoreceptors and lesions borders. Regions bordering areas of atrophy are shown here for all 5 subjects who were imaged using both confocal and split-detector AOSLO. Confocal images of 400 x 400 μm regions of interest (ROIs) are shown beside split-detector images of the same areas. Subjects and modalities are labeled. Confocal and split-detector images from a normal male, JC_0616, are shown for comparison. The ROIs are located at the following eccentricities: KS_0044, 3780 μm; JC_0699, 1432 μm; JC_0778, 2772 μm; JC_0782, 3050 μm; DW_10173, 1883 μm; JC_0616, 1899 μm. Note that confocal images often present confounding ambiguities in pathology: lesion borders are poorly defined, and abnormal cone reflectivity cannot be clearly distinguished from the reflectivity arising from rods, debris, and/or atrophic retina. In contrast, split-detector images offer superior delineation of lesion borders and unambiguous imaging of cone inner segments. Rods can be seen between cones in 4 of 5 cases (all but JC_0699). **Scale bar:** 100 μm.

**Fig 2** shows a comparison of confocal and split-detector AOSLO images in all 5 subjects imaged with both modalities and one healthy control subject. The split-detector modality provides unambiguous visualization of cone inner segments in areas of preserved retina and near lesion borders. While increased scleral shine-through on confocal AOSLO imaging can be used to approximate lesion borders, split-detector imaging can more precisely identify and localize lesion borders as sharply-circumscribed perimeters where photoreceptor inner segment mosaics terminate. Furthermore, confocal AOSLO cannot be used to reliably identify cones in far-peripheral or pathologic areas due to irregularities in confocal reflectivity, where some cones appear as clusters of bright spots not easily distinguishable from rod or RPE reflectivity. In contrast, split-detector AOSLO unambiguously resolves cone inner segments even in the very peripheral and/or diseased areas, enabling observation of pathologic cone density, size, and morphology.

### Sharp Transitions Observed Between Areas with Remnant Cone Mosaic and Areas of Atrophy

**Fig 3:**
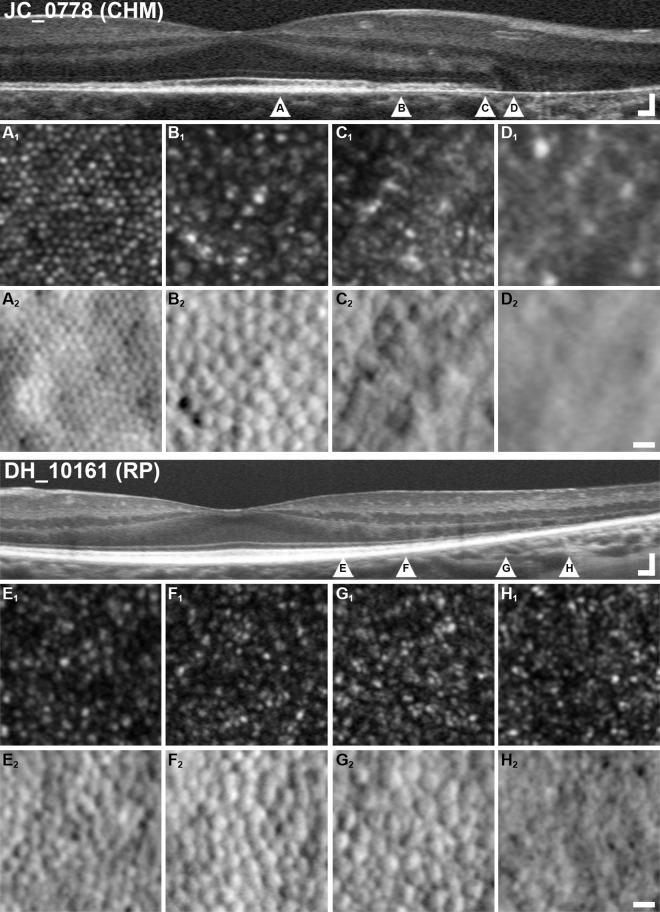
Abrupt termination of photoreceptor mosaic at lesion borders in choroideremia is contrasted to gradual loss of photoreceptors in retinitis pigmentosa. Images from a subject with X-linked choroideremia (JC_0778, **top**) are compared with images from a previously-published[32] subject with retinitis pigmentosa (DH_10161, **bottom**). To facilitate comparison, images of the same modality share the same scaling. Labeled **arrowheads** in OCTs correspond to respectively labeled AOSLO images, and imaged regions are selected to correspond to pathologic features. Panels **A** and **E** show 0.25 x 0.25° (approximately 74 x 74 μm) areas of retina with intact IZ and EZ bands on SD-OCT. In both pathologies, cone inner segments appear grossly normal on split-detector (**A**_**2**_**, E**_**2**_), while only RP displays notably dim or dark cones on confocal (**E**_**1**_). Panels **B** and **F** show areas of retina with IZ band dropout but intact EZ. Confocal AOSLO in both (**E**_**1**_**, F**_**1**_) shows some irregular “multimodal” reflectivity, with dark cones again visible in the RP subject (**F**_**2**_). Split-detector imaging (**E**_**2**_**, F**_**2**_) again shows relatively normal inner segments. Panels **C** and **G** correspond to IZ and EZ dropout. In choroideremia, EZ dropout coincides closely with the loss of the ELM and presence of an interlaminar bridge (ILB). On AOSLO, there is an abrupt termination to the photoreceptor mosaic (**C**_**2**_), with no cone inner segments present past this point (**D**_**2**_). In RP, ILBs are generally not seen, and the ELM continues well past EZ dropout. On AOSLO, remnant cone inner segments similarly persist well past the end of the EZ (**G**_**2**_), finally disappearing at or near the point of ELM termination (**H**_**2**_). **Scale bars: OCTs,** 100 μm lateral, 100 μm axial; **AO,** 10 μm.

We observed variable photoreceptor density, spacing and morphology near lesion borders and abrupt delineations between areas with a remnant cone mosaic and areas of atrophy. This does not seem to be the case in other retinal dystrophies such as retinitis pigmentosa (RP), in which the transition between preserved and atrophic retina occurs over an extended transition zone several hundreds or thousands of microns wide.[32] **Fig 3** contrasts SD-OCT and AOSLO retinal imaging in a representative subject (JC_0778) with choroideremia with previously-published[32] SD-OCT and AOSLO images from a subject (DH_10161) with RP. Although DH_10161 demonstrates a classic pattern of peripheral-to-central degeneration, it should be emphasized that RP is an extremely diverse family of diseases with equally broad-ranging findings that cannot be fully encompassed by a single subject.

In 4 of the 5 choroideremia subjects for whom both confocal and split-detector imaging were available, outer segment reflectivity (seen on confocal AOSLO) and inner segment structure (seen on split-detector AOSLO) were simultaneously extinguished at lesion borders. The cone inner segment mosaic in areas of preserved retina formed a largely continuous and closely packed mosaic up to the edge of the lesion, but was observed to abruptly terminate at the point of EZ dropout (**Fig 3C**). This point of EZ dropout and inner segment disappearance also coincided with ELM dropout in these 4 cases, and with an interlaminar bridge (ILB) in at least one direction in these 4 cases, consistent with reports by Lazow et al.[9] In the 5^th^ case, the cone mosaic termination point was beyond the SD-OCT scan volume.

Foveal cone reflectivity was relatively normal in subjects with choroideremia (**Fig 3A**_**1**_), which differed from the numerous non-waveguiding cones visible in subjects with RP even in areas of intact IZ (**Fig 3E**_**1**_).[32] In both choroideremia and RP, the inner segment mosaic remains densely and regularly packed in pre-IZ dropout areas. Finally, in both conditions, the cone mosaic exhibits dimmer and altered confocal reflectivity after IZ band dropout on SD-OCT, but appears largely normal on split-detector AOSLO (**Fig 3B**).

### Heterogeneous Photoreceptors and Photoreceptor Mosaics at Lesion Borders

**Fig 4:**
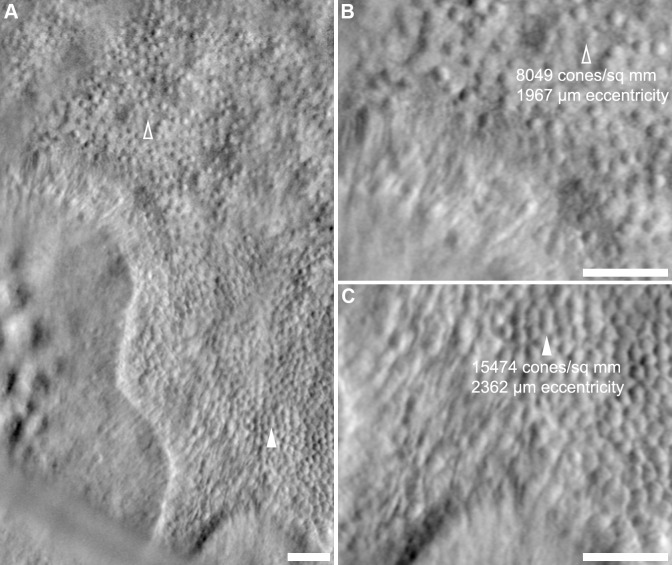
Variability in photoreceptor density near lesion borders. **Panel A** displays a border region from KS_0044 approximately 1° nasal from the area shown in **Fig 2**, located at approximately 6.5° to 8.5° eccentricity. **Arrowheads** indicate two regions within ~100 μm of the lesion edge; the cone mosaic at the **open arrowhead** is noticeably sparser (**B**, 8,049 cones/mm^2^) than at the **closed arrowhead** (**C**, 15,474 cones/mm^2^), which is approximately 395 μm (or approximately 1.36°) more eccentric from the fovea. For comparison, normal mean ± standard deviation cone density measured from 9 normal subjects at an eccentricity of 7.36° (~2,142 μm) is 11,833 ± 1,816 cones/mm^2^.[36] **Scale bars:** 50 μm.

Cone density in approximately equally-eccentric areas of remnant retina near the edges of atrophic lesions was observed to vary significantly in all 5 subjects imaged with split-detector AOSLO (**Fig 4**). To sample this variability, paired cone-counting ROIs in areas of high and low density were manually selected in each montage. Split-detector images were used, as cone densities could not be reliably measured near lesion borders using confocal AOSLO. The mean eccentricity difference between sampling ROIs for high- vs. low-density areas of the mosaic was 186 μm (maximum eccentricity difference = 395 μm), and ROIs were cropped within 200 μm of the nearest lesion border. Complete results are presented in **Table 2**.

**Table 2:** Cone Density Variability Near Lesion Borders.

Subject	Dense Region		Sparse Region		Expected Density Difference	Measured Density Difference	Mean Normal Data		
	Density (Cones/mm^2^)	Eccentricity (mm)	Density (Cones/mm^2^)	Eccentricity (mm)			Mean Density (Cones/mm^2^)	Standard Deviation	Eccentricity (mm)
**KS_0044**	15,474*	2.362	8,049^†^	1.967	-1,636	7,425^‡^	11,833	1,816	2.142
**JC_0699**	17,960	1.725	9,937	1.658	-383	8,023^‡^	14,122	2,098	1.694
**JC_0778**	5,917	3.256	5,276	3.529	712	641	7,305^§^	1,506^§^	3.455^§^
**JC_0782**	15,062*	3.684	10,458*	3.646	-91	4,604^‡^			
**DW_10173**	16,857	1.222	10,152^†^	1.066	-2,034	6,705^‡^	19,966	3,226	1.127

Expected density difference = (mean normal cone density at eccentricity of dense region in choroideremic patient)–(mean normal cone density at eccentricity of sparse region in choroideremic patient). Negative value indicates the region with denser cones was actually expected to have sparse cones due to greater eccentricity from the fovea.

* Cone density is >2 SD greater than normal mean cone density at a comparable eccentricity.

† Cone density is >2 standard deviations (SD) less than normal mean cone density at a comparable eccentricity.

‡ Cone density difference is >2 SD of normal mean cone density at a comparable eccentricity.

§ The same measured normal data was used in comparison to data from both JC_0778 and JC_0782 because of similar eccentricities of measurements.

In one case (JC_0778), the measured difference in cone density was similar to the expected calculated normal density difference due to change in eccentricity (see Methods). In the remaining 4 cases, measured density differences deviated greatly from expected density differences. Furthermore, in all subjects except JC_0778, cone density variability between paired ROIs exceeded 2 SD of the mean cone density measured from 9 normal subjects.[36] The mean difference in eccentricity between normal and choroideremia sampling areas was 63 μm (maximum eccentricity difference = 210 μm). Interestingly, cone densities could be either significantly greater than (JC_0782) or less than (DW_10173) normal. One subject, KS_0044, had cone densities both statistically significantly greater and lower than normal.

### Axial Localization of Bubble-like Lesions

**Fig 5:**
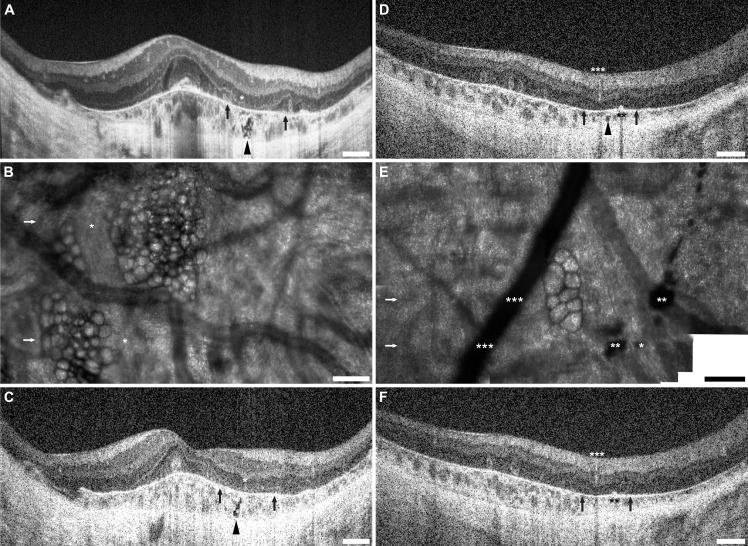
Bubble lesions correspond to subretinal hyporeflective regions on SD-OCT. SD-OCT and confocal AOSLO imaging of bubble lesions are presented here from two subjects, JC_0618 (**left**) and JC_0752 (**right**). The lateral distance subtended by the AOSLO imaging windows are indicated by two **black arrows** in frames **A, C, D,** and **F,** while the location of the SD-OCT B-scans are indicated by **white arrows** in frames **B** and **E**. In JC_0618, an ORT is indicated by the asterisk (*) allowing mapping of the bubble-like lesions seen in **B** to the regions indicated by **black arrowheads** in **A** and **C**. In JC_0752, double asterisks (**) indicate pigment clumps, while triple asterisks (***) mark a retinal blood vessel. These features help localize the bubble-like lesions in **E** to the region indicated by the **black arrowhead** in **D**. In **F**, where the plane of the B-scan does not cross the bubble-like lesions, and the spot in **D** is no longer visible. **Scale bars: A, C, D** and **F**, 500 μm; **B** and **E**, 100 μm.

Bubble-like lesions were frequently observed on confocal AOSLO in the atrophic retina near lesion borders. Previous reports have proposed that these lesions may correspond to subretinal structures seen on SD-OCT.[26] Findings in our subjects concur with this observation. After SD-OCT images were precisely scaled, aligned, and mapped to confocal AOSLO montages, nearby objects of interest (*e*.*g*. retinal tubulations, pigment clumps, and blood vessels) were used to assist localization of bubble-like lesions to the approximate areas labeled by black arrowheads on SD-OCT (**Fig 5**).

### Remnant Cone Structure within Interconnected Outer Retinal Tubulations (ORTs)

**Fig 6:**
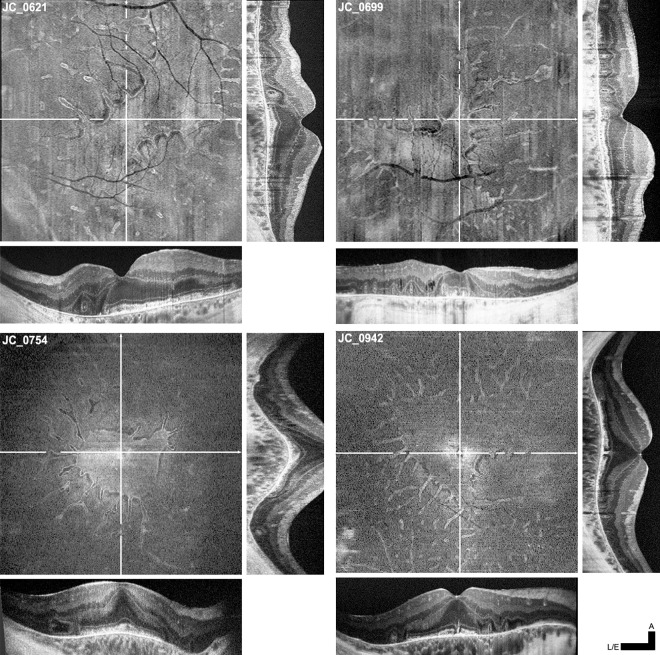
ORTs viewed *en face* on SD-OCT are contiguous with central regions of preserved retina. Four sets of SD-OCT *en face* and B-scan images are presented here for subjects JC_0621, JC_0699, JC_0754, and JC_0942. *En face* projections are aligned to averaged line scans passing horizontally (**bottom**) and vertically (**right**) through the fovea. **Orthogonal lines** on each *en face* OCT indicates the locations at which line scans were taken. B-scan cross sections of ORTs can be seen as distinct hyperreflective ovaloid structures superficial to Bruch’s membrane; in *en face* projection, ORTs appear as long, thin protrusions. Most ORTs are contiguous with the central region of preserved retina, though remnant “island” ORTs can also be seen amidst surrounding atrophy. **Scale bars:** all lateral & *en face* OCTs (L/E) = 1,000 μm, axial OCTs = 200 μm.

Outer retinal tubulations (ORTs) were observed in 10 of 12 subjects on SD-OCT. **Fig 6** shows representative *en face* SD-OCT images alongside averaged horizontal and vertical line scans through the fovea. Consistent with previous reports,[13, 14, 30] ORTs typically appear on SD-OCT as ovaloid hyperreflective structures with hyporeflective lumens located between the RPE and the outer nuclear layer. IZ and EZ bands were not discernible within ORTs. The axially elongated shape of ORTs is an artifact of SD-OCT aspect ratio, which is typically chosen to provide higher axial vs. lateral magnification. Histology has shown that ORTs are more frequently axially flattened ovaloids.[14] Viewed *en face*, these ORTs can be clearly seen as long, thin protrusions that are mostly interconnected and contiguous with central preserved retina. Some “island” ORTs were observed, which may have detached from nearby ORTs through progressive degeneration (**Fig 6**).

**Fig 7:**
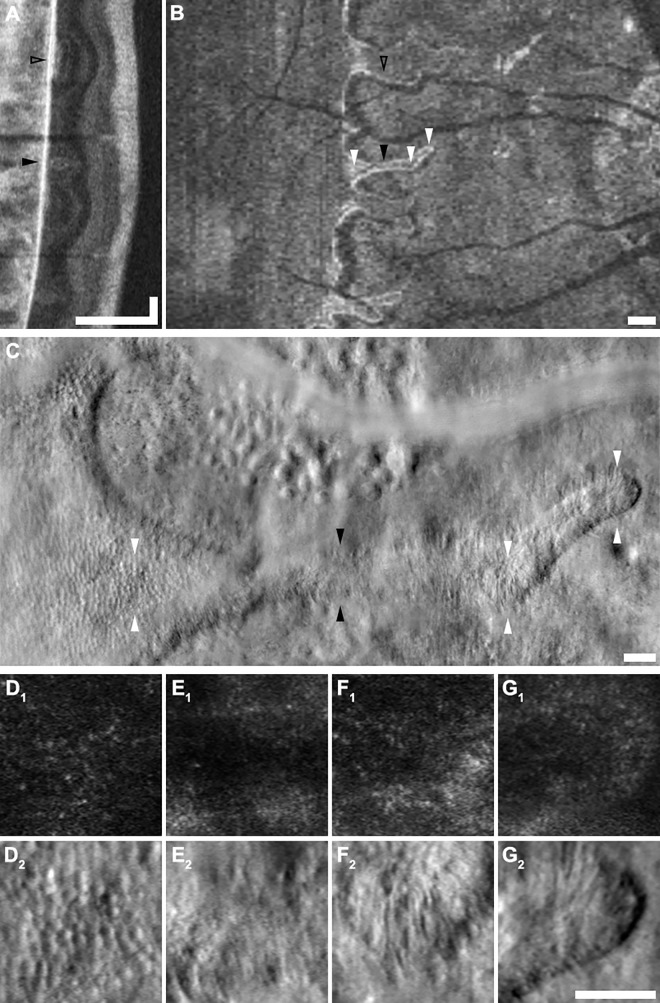
Multimodal imaging of ORTs. **A-G** present a vertical SD-OCT B-scan (**A**), an *en face* SD-OCT projection (**B**), a split-detector AOSLO montage (**C**), and zoomed-in views of the montage (**D-G**) of an ORT in subject JC_0778. **Labeled arrowheads** indicate corresponding locations in different modalities. The ORT indicated by the **filled black arrowhead** is structurally distinct on B-scan (**A**) from the wider patch of preserved retina just superior (indicated by the **open black arrowhead**). **Top** and **bottom** arrowheads in **C** indicate the top and bottom edges of the zoomed-in 100 x 100 μm images provided by panels **D** to **G**. Relatively normal cones are seen in the region of preserved retina (**D**) while sparse, morphologically abnormal remnant cone inner segments are seen throughout the ORT (**E, F, G**). **Scale bars: A,** lateral (vertical) & axial (horizontal) = 250 μm, axial (horizontal) = 100 μm; **B,** 250 μm; **C,** 50 μm; **D-G,** 50 μm.

By precisely aligning SD-OCT B-scans, SD-OCT *en face* projections, and AOSLO confocal and split detector images, direct comparison of the anatomy of preserved retina and ORTs across different imaging modalities was possible. Of the 5 subjects imaged with split-detector AOSLO, 4 displayed ORTs (JC_0782 had relatively mild disease and no ORTs were visualized). **Fig 7** shows an example comparison of the same ORT and an immediately adjacent area of remnant retina across multiple imaging modalities. SD-OCT imaging reveals that the infolded ORT is morphologically distinct from the nearby, wider ORT-like region of preserved retina, in which the typical retinal architecture is attenuated but partially retained.

In the split-detector images, large, sparse, remnant cone inner segments were clearly observed within ORTs of all subjects. **Panels 7D**_**2**_**-G**_**2**_ show split-detector AOSLO images of representative ORTs from these 4 subjects. Cone inner segments of relatively normal diameter, density and orientation are also seen in adjacent areas of preserved retina. The split-detector imaging wide-field montage of the border of an area of atrophy in KS_0044 (**Fig 7C**) reveals most of the salient features discussed in preceding sections, including abnormal cone mosaics, ORTs, and bubble lesions.

## Discussion

The recently-developed split-detector AOSLO imaging modality allows unambiguous imaging of cone photoreceptor inner segments even in the setting of pathologies that disrupt waveguiding.[28–33] This circumvents an inherent drawback of previous AO imaging modalities in which photoreceptor visualization was dependent on the waveguided reflectance of imaging light through an intact cone outer segment.[28]

Using split-detector AOSLO imaging, we examined the photoreceptor mosaic in subjects with choroideremia and uncovered several novel findings. Foveal cone densities were normal in subjects with preserved EZ bands on SD-OCT, though it should be noted that disease progression was relatively mild in these patients. The cone mosaic was contiguous throughout regions of preserved retina; however, cone densities in areas near lesion borders could be significantly increased or decreased from normal, and individual cone inner segments appeared dysmorphic and enlarged. The loss of cone reflectivity on confocal AOSLO at lesion borders (previously reported by Morgan et al.[26]) was found to colocalize with a complete loss of the remnant cone inner segment mosaic and with ILBs. This sharp termination of the cone inner segment mosaic at well-delineated lesion borders, combined with early IZ band abnormalities and RPE band thinning on SD-OCT,[26] reinforces the theory that cone loss is predicated on underlying RPE degeneration, and may offer additional evidence toward a primary RPE degeneration model of choroideremia.[6, 8, 12, 26]

The macro- and microanatomy of ORTs was also examined. Zweifel et al.[13] first reported these characteristic structures in OCT images acquired from subjects with age-related macular degeneration,[13] while recent work by Litts et al. revealed that the branching, interconnected structure of ORTs is composed of degenerating cones and infolded ELM.[14] Consistent with recent results published by Litts *et al*.,[27] ORTs did not display IZ/EZ bands on SD-OCT or recognizable cone reflectivity on confocal imaging. However, we did observe remnant cone inner segments in these structures using split-detector imaging. These cones appeared enlarged and morphologically irregular, suggesting a state of advanced degeneration. Interestingly, this association of remnant inner segments to preserved ELM is also seen in the transition zone of retinitis pigmentosa. However, in choroideremia, the ELM typically terminates too close to EZ dropout to definitively associate its presence with inner segment visibility.[32] Our data also confirms that although noncontiguous ORT “islands” could be seen near ORT tips, ORTs are generally contiguous with one another and with the central region of preserved retina. As was previously proposed,[13] ORTs appeared to degenerate slowly, persisting relatively unchanged over several months or years of follow-up imaging. Together, these findings suggest that ORTs are an end stage of retinal degeneration.

Finally, we endeavored to localize the “bubble-like” lesions previously noted by Morgan et al.[26] These lesions were best seen on confocal AOSLO, and by precisely aligning these images with SD-OCT of the same area, these lesions were mapped to choroidal sources on SD-OCT. However, it should be emphasized that the exact source of these bubble-like lesions has yet to be determined, owing both to a relatively large depth of field on AOSLO and limited resolution on SD-OCT. Furthermore, these structures cannot be readily identified by SD-OCT alone at this point, as they are not easily distinguished from other structures in the choroid (*e*.*g*., blood vessels). Although more work is needed to characterize the pathophysiology of these lesions, bubble-like lesions were most frequently seen in areas near the leading edge of atrophic lesions, possibly suggesting a link to recent or active atrophy.

This study illustrates some of the benefits of a multimodal imaging approach combining SD-OCT with confocal and split-detector AOSLO in studying retinal degenerations, and contributes to a growing body of literature concerning the pathophysiology and progression of choroideremia. In the future, we expect additional studies using these modalities, as well as emerging techniques including AO microperimetry,[51] OCT-angiography and directional OCT,[52] will provide further insight into choroideremia and other retinal degenerations. In addition, these non-invasive, *in vivo* high-resolution imaging modalities may aid in the selection of eligible subjects for novel gene therapy trials[34] and offer improved monitoring of disease progression.

## Supporting Information

S1 Fig Vertical SD-OCTs of all 12 subjects with X-linked choroideremia. Shown here are vertical SD-OCT line scans through the fovea of all 12 subjects (labeled) taken concurrently with the SD-OCTs presented in **Fig 1.** Images are scaled and cropped to subtend a uniform retinal distance (6 mm). ORTs are labeled with **filled arrowheads**, and ILBs are labeled with **open arrowheads**. **Scale bars, axial & lateral:** 250 μm.
(TIF)

S2 Fig Sampling windows used for near-foveal cone mosaic density analyses. Cone densities were measured at a mean 0.70° (202.5 μm) eccentricity in 5 subjects with intact foveal EZ bands on SD-OCT. The confocal AOSLO regions of interest (ROIs) used in cone density analyses are shown here with subject IDs superimposed for identification. A normal subject (JC_0616) has been included for comparison. **Scale bar:** 10 μm.
(TIF)
