# Supplementary figures and images for: Multimodal Imaging of Photoreceptor Structure in Choroideremia
Source: PLoS One. 2016 Dec 9;11(12):e0167526. doi: 10.1371/journal.pone.0167526 (PMC5147929; doi:10.1371/journal.pone.0167526)

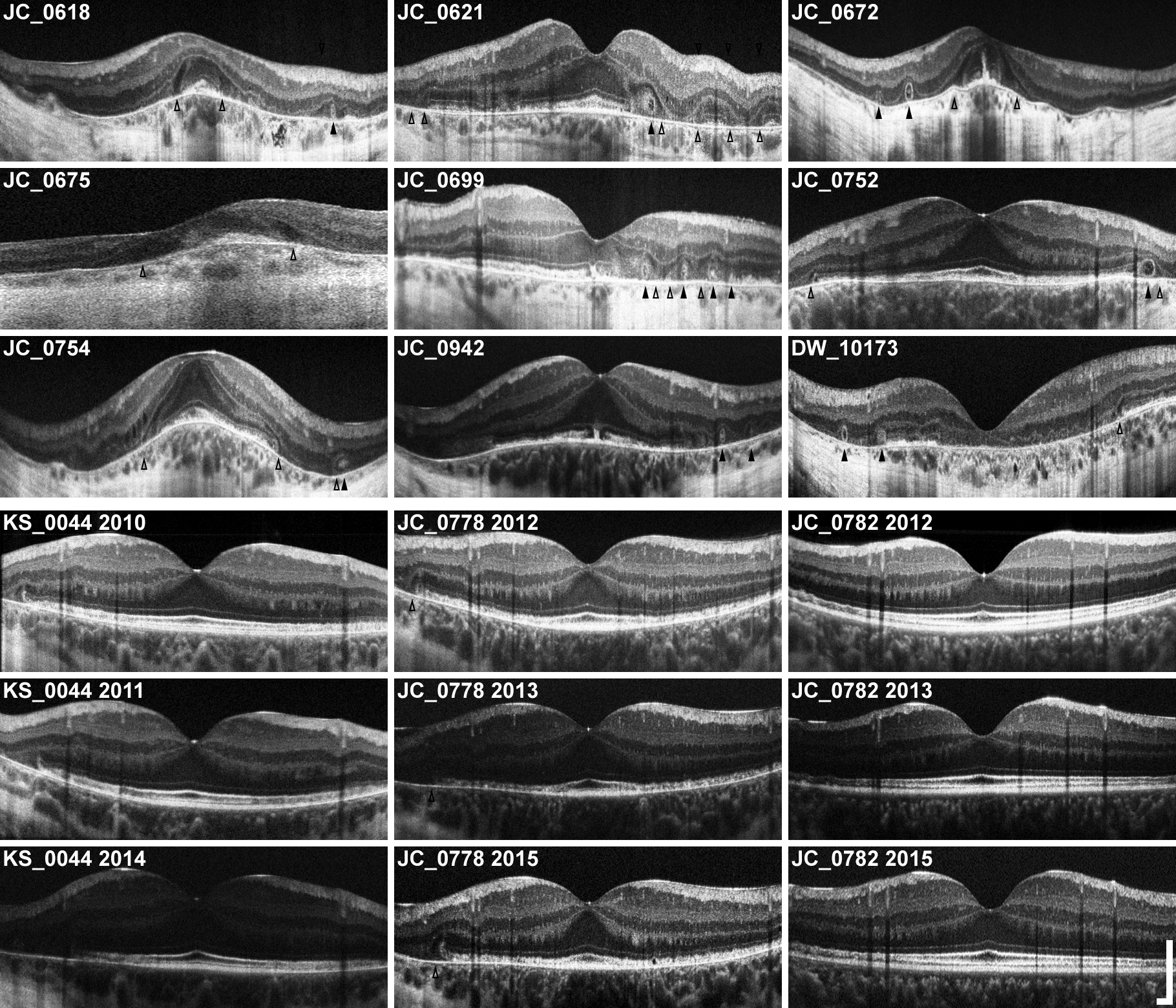

Supplement: S1 Fig — Shown here are vertical SD-OCT line scans through the fovea of all 12 subjects (labeled) taken concurrently with the SD-OCTs presented in Fig 1. Images are scaled and cropped to subtend a uniform retinal distance (6 mm). ORTs are labeled with filled arrowheads, and ILBs are labeled with open arrowheads. Scale bars, axial & lateral: 250 μm. (TIF) [file pone.0167526.s001.tif]

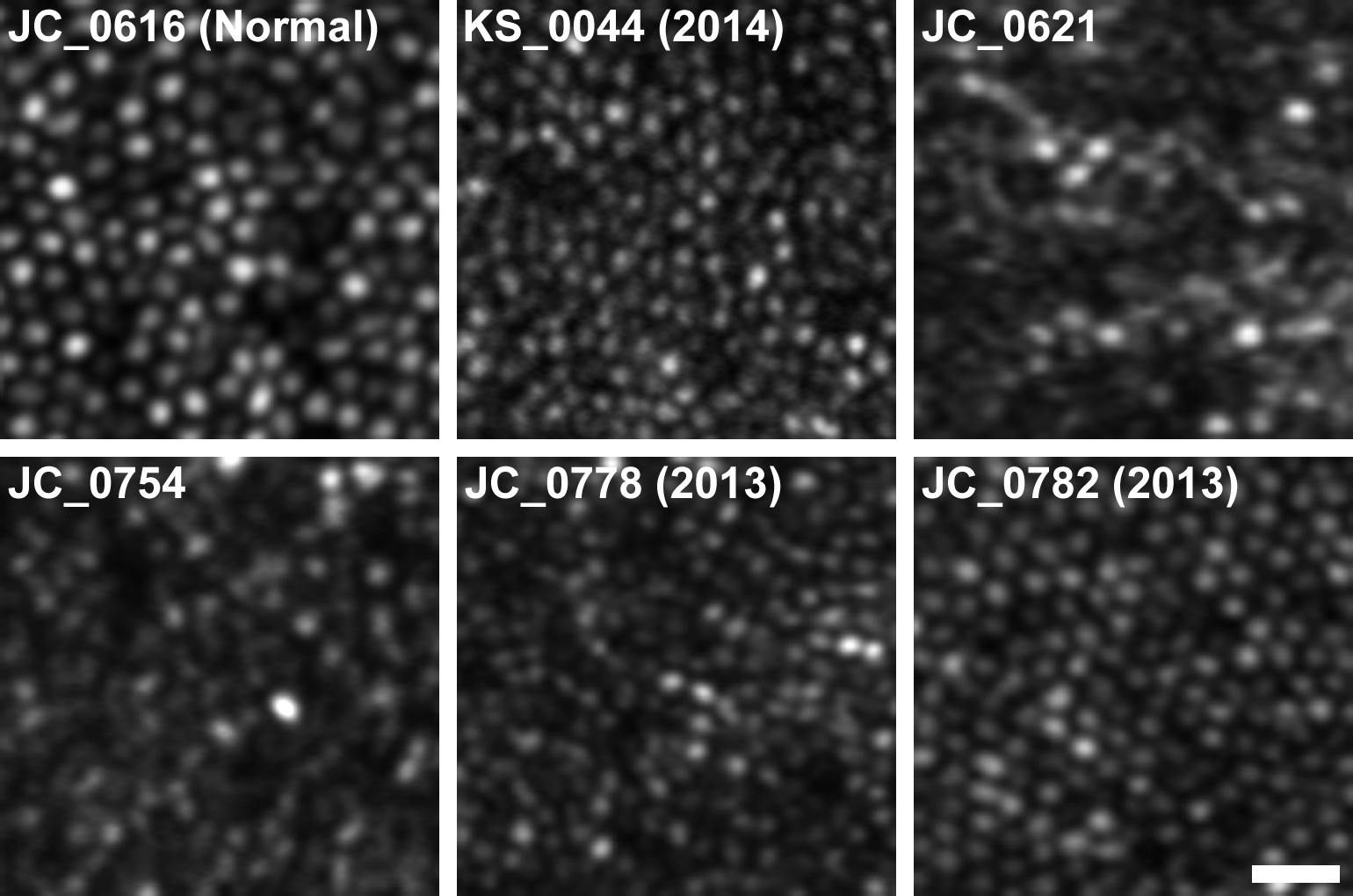

Supplement: S2 Fig — Cone densities were measured at a mean 0.70° (202.5 μm) eccentricity in 5 subjects with intact foveal EZ bands on SD-OCT. The confocal AOSLO regions of interest (ROIs) used in cone density analyses are shown here with subject IDs superimposed for identification. A normal subject (JC_0616) has been included for comparison. Scale bar: 10 μm. (TIF) [file pone.0167526.s002.tif]
